# HPV-Related Disease: Gynecologists’ Leadership in Patient-Centered Multidisciplinary Care

**DOI:** 10.3390/cancers18132116

**Published:** 2026-06-30

**Authors:** Nicolò Clemente, Giovanni Delli Carpini, Jacopo Di Giuseppe, Luca Giannella, Francesco Sopracordevole, Leonardo Natalini, Antonino Ditto, Andrea Ciavattini, Italian Society of Colposcopy and Cervico-Vaginal Pathology (SICPCV)

**Affiliations:** 1 Gynecological Oncology Unit, Centro di Riferimento Oncologico di Aviano (CRO), IRCCS, National Cancer Institute, Via Franco Gallini 2, 33081 Aviano, Italy; nicolo.clemente@cro.it (N.C.); antonino.ditto@cro.it (A.D.); 2 Obstetrics and Gynecologic Section, Department of Odontostomatologic and Specialized Clinical Sciences, Università Politecnica delle Marche, Via F. Corridoni 11, 60123 Ancona, Italy; giovanni.dellicarpini@ospedaliriuniti.marche.it (G.D.C.); jacopo.digiuseppe@ospedaliriuniti.marche.it (J.D.G.); luca.giannella@ospedaliriuniti.marche.it (L.G.); l.natalini@pm.univpm.it (L.N.); 3 Obstetrics and Gynecologic Unit, “Ca’ Foncello” Hospital, Piazzale Ospedale 1, 31100 Treviso, Italy; drfrancesco@sopracordevole.it

Human papillomavirus (HPV) is the most common sexually transmitted infection worldwide, with more than 80% of women and 90% of men expected to acquire the infection at some point during their lifetime [1]. Globally, HPV is responsible for approximately 4.5% of all cancers in both women and men [2]. High-risk HPV (HR-HPV) types 16 and 18, together with a limited group of additional genotypes (e.g., 31, 33, 45, 52, 58), account for the vast majority of HPV-related malignancies [3]. Cervical cancer (CC) remains the single largest contributor, representing roughly 80% of HPV-related cancers: estimates indicate 604,000 new CC cases and 342,000 deaths in 2020, rising to about 662,000 cases and 349,000 deaths in 2022, with a disproportionate burden in low- and middle-income countries [4,5]. The substantial geographical variation in CC incidence and survival is largely driven by differences in the availability of primary and secondary prevention strategies [6].

Prophylactic HPV vaccination has been extensively evaluated and consistently shown to be highly effective in preventing persistent infection with HR-HPV and the development of cervical precancerous lesions, which are necessary precursors to invasive CC [6].

Prophylactic HPV vaccination is therefore recommended in both females and males as a public health measure to reduce HPV circulation and transmission. Broader vaccination coverage is expected to contribute to herd immunity and to the overall reduction in HPV-related diseases [6,7]. In recognition of these strong evidences, multiple international and national health authorities and medical societies, including the World Health Organization (WHO), the US Centers for Disease Control and Prevention (CDC) and Advisory Committee on Immunization Practices (ACIP), the American Society of Colposcopy and Cervical Pathology (ASCCP), the European Federation of Colposcopy (EFC) with the European Society of Gynaecological Oncology (ESGO), and the Italian Society of Colposcopy and Cervico-Vaginal Pathology (SICPCV), have already published position papers and guidelines emphasizing both HPV vaccination and HPV primary screening as central strategies for primary prevention of CC [7].

HPV vaccination has also been evaluated as a potential strategy to reduce the risk of recurrent cervical dysplastic lesions in adult patients undergoing cervical conization for high-grade cervical intraepithelial neoplasia (CIN) [8]. Although some studies suggest a possible benefit, the available evidence is derived predominantly from non-randomized intervention studies with serious or critical risk of bias and low to very low certainty. However, evidence from randomized controlled trials (RCTs) remains limited [9,10].

Beyond the cervix, HPV drives a broad spectrum of anogenital and oropharyngeal malignancies. Persistent HR-HPV infection is causally linked to the majority of anal cancers (ACs), as well as a large fraction of vulvar, vaginal, and penile cancers (PCs) and oropharyngeal squamous cell carcinoma (OPSCC) [6]. Interestingly, in high-income settings, the HPV-attributable cancer profile is shifting: while CC incidence is slowly declining in many countries with organized screening and vaccination programs, HPV-related extracervical cancers such as AC and OPSCC have risen sharply; the latter being related to different attitudes toward oral sex in different countries [11,12,13,14]. This evolving pattern underscores that HPV is no longer a concern confined to the cervix but a multisite oncogenic infection affecting both women and men across the anogenital tract and the upper aerodigestive tract.

Although prophylactic HPV vaccination has demonstrated high efficacy in preventing persistent HPV infection and high-grade CIN, resulting in a substantial reduction in the incidence of invasive CC, its observed impact on extracervical HPV-related lesions appears to be more modest and variable, largely due to their lower baseline incidence [6]. Nonetheless, based on biological plausibility and shared oncogenic pathways, HPV vaccination is expected to also reduce the risk of other HPV-related anogenital and oropharyngeal cancers. For this reason, sustained efforts should be made to improve HPV vaccination coverage in both female and male populations.

Furthermore, no consensus guidelines on the screening of extracervical HPV-related conditions exist. For example, there are no standard routine screening tests for PC in the general population, although early detection, diagnosis, and treatment are crucial for improving prognosis [15].

Similarly, to date, there are no reliable tests for HPV screening in patients with OPSCC, although some studies reported interesting results [16].

In contrast, targeted AC screening programs have been developed for specific high-risk subgroups of the population, including individuals living with Human Immunodeficiency Virus (HIV), men who have sex with men (MSM), transgender women (TW), women with previous HPV-related vulvar intraepithelial neoplasia (vulvar HSIL) or vulvar cancer (VC) and immunocompromised patients, in whom the incidence of anal cancer and its precursors is substantially higher than in the general population [17]. In these high-risk populations, anal cytology and high-resolution anoscopy in cases of cytological abnormalities are recommended [17].

In the context of the increasing global burden of HPV-related disease, gynecologists, and especially colposcopists, play a pivotal role in shaping and implementing comprehensive cancer prevention strategies that address both genital and extragenital HPV-associated malignancies. Through their involvement in secondary prevention via organized screening programs and patient education across the life course, gynecologists are uniquely positioned to promote an integrated approach to HPV-related cancer control.

Comprehensive, accurate, and empathetic counseling represents a cornerstone of this integrated, multidisciplinary management of HPV-related disease. Counseling should address not only medical aspects but also the psychosexual and relational implications of HPV infection within the couple. The following key concepts should be systematically addressed during counseling:

- HPV infection is highly prevalent and primarily involves the anogenital tract; however, it may also affect other anatomical sites, including the oropharynx;
- Prophylactic HPV vaccination should be encouraged because it is highly effective in preventing persistent infection with HR-HPV and the development of cervical precancerous lesions, thereby reducing the incidence of invasive CC;
- Although currently available data remain limited, prophylactic HPV vaccination may potentially reduce the risk of other HPV-related anogenital and extragenital cancers; therefore, it should be encouraged in both females and males;
- Most sexually active individuals will acquire HPV infection at some point during their lifetime, often transiently and without clinical consequences;
- Sexual partners frequently share HPV infection, and it is not possible to determine the timing or direction of transmission. The presence of HPV infection does not imply recent transmission or sexual activity outside the current relationship;
- HPV testing of the sexual partners of women with HPV infection or precancerous cervical lesions is not recommended, as it provides no clinical benefit and does not influence management;
- Female partners of men who report a previous female partner with HPV infection should undergo CC screening according to standard population-based intervals; there is no evidence that intensified screening confers additional benefit;
- Smoking cessation should be strongly recommended, given the well-established association between tobacco exposure and the persistence and progression of HPV-related precancerous and malignant lesions;
- Anogenital warts, although caused by low-risk HPV types, are highly contagious; therefore, sexual partners should be appropriately informed. Because HPV infection is often already present, even in the absence of visible lesions, HPV testing of partners is not indicated;
- Routine peniscopy is not recommended for asymptomatic sexual partners of women with HPV infection or precancerous cervical lesions. However, it may be considered in partners of women with clinically evident genital warts. Peniscopy should be considered in the presence of suggestive signs or symptoms, including visible penile lesions (such as warts, papules, plaques, or erythematous or pigmented areas), persistent pruritus, burning or discomfort, pain, bleeding, ulceration, or other unexplained penile skin or mucosal changes;
- When indicated, peniscopy should be performed by an experienced colposcopist or by another healthcare professional, such as a urologist or dermatologist, with specific expertise in the detection and management of penile intraepithelial lesions;
- Identification of high-risk subgroups is essential to recognize individuals at increased risk of AC who may benefit from anal cytological screening within specialized settings;
- AC screening is particularly recommended in HIV-positive individuals, MSM, TW, women with a history of vulvar HSIL or HPV-related VC, and solid organ transplant recipients;
- Women with a history of high-grade CIN or VaIN, or the corresponding carcinomas, individuals with perianal condylomata, those with persistent HPV 16 infection, and patients with autoimmune diseases may benefit from AC screening starting from the age of 45 years, following shared decision-making;
- Routine screening of the oral cavity and oropharynx is currently not feasible, as no identifiable precancerous lesions or validated screening strategies exist. Furthermore, currently available HPV molecular assays are not validated for anatomical sites other than the cervix and vagina;
- In the presence of persistent or suspicious symptoms (e.g., prolonged sore throat, otalgia, hoarseness, cervical lymphadenopathy, or odynophagia), prompt referral to an otorhinolaryngology specialist is recommended.

HPV-related disease represents a multisite oncogenic condition that extends beyond cervical pathology and requires a patient-centered, multidisciplinary approach to prevention, screening, diagnosis, treatment, and long-term follow-up. In this evolving landscape, gynecologists, and particularly colposcopists, play a central role, bridging primary and secondary prevention strategies, coordinating care across specialties, and providing comprehensive counseling throughout the patient’s life course. Increasing awareness of extragenital HPV-associated malignancies and strengthening multidisciplinary collaboration are essential to optimizing clinical outcomes, reducing disparities in the HPV-related cancer burden, and addressing the medical, psychological, and relational impact of HPV infection. In this context, the development of centralized, structured multidisciplinary care pathways for patients with HPV infection is crucial to ensure timely access to expertise, continuity of care, and consistent, high-quality management across the disease spectrum.

## Abbreviations

The following abbreviations are used in this manuscript:

AC	Anal Cancer
CC	Cervical Cancer
CIN	Cervical Intraepithelial Neoplasia
HIV	Human Immunodeficiency Virus
HPV	Human Papillomavirus
HR-HPV	High-Risk Human Papillomavirus
HSIL	High-grade Squamous Intraepithelial Lesion
MSM	Men who have Sex with Men
OPSCC	Oropharyngeal Squamous Cell Carcinoma
PC	Penile Cancer
RCT	Randomized Controlled Trial
TW	Transgender Women
VaIN	Vaginal Intraepithelial Neoplasia
VC	Vulvar Cancer
